# Phenotypic Diversity of Doum Palm *(Hyphaene compressa),* a Semi‐Domesticated Palm in the Arid and Semi‐Arid Regions of Kenya

**DOI:** 10.1155/2020/4920830

**Published:** 2020-12-29

**Authors:** Agnes Omire, Nancy L. M. Budambula, Johnstone Neondo, Robert Gituru, Cecilia Mweu

**Affiliations:** ^1^Department of Botany, Jomo Kenyatta University of Agriculture and Technology, P. O. Box 62000-00200, Nairobi, Kenya; ^2^Department of Biological Sciences, University of Embu, P. O. Box 6-60100, Embu, Kenya; ^3^Institute for Biotechnology Research, Jomo Kenyatta University of Agriculture and Technology, P. O. Box 62000-00200, Nairobi, Kenya

## Abstract

*Hyphaene compressa* is an economically important palm in Africa. Despite its significant role in the livelihoods of rural communities, the diversity of doum palm is poorly documented and studied. In addition, it has no model descriptor that can aid such studies. Ninety *H. compressa* accessions collected from Northern, Eastern, and Coastal regions of Kenya were examined to determine the morphological variability of the vegetative and fruit traits of *H. compressa* and to identify its morphotypes for improvement. A total of 19 morphological characters including seven quantitative and 12 qualitative traits of fruit and vegetative traits were selected. Linear mixed-effects models, principal component analysis, and linear discriminant analyses were used to assess the variation in the morphological traits of doum palm based on the regions. Hierarchical clustering was performed to identify the morphotypes of *H*. *compressa*. There was variability in *H*. *compressa* morphological traits, particularly at the Kenyan Coast. All seven quantitative traits were able to effectively discriminate doum palm phenotypically (*p* ≤ 0.001). The 90 accessions clustered into five morphotypes designated as 1, 2, 3, 4, and 5. Morphotype 4 was specific only to the Coastal region. Morphotype 5 had the tallest trees with the biggest fruits and included palms from Eastern and Coastal regions making it the best morphotype for fruit traits. This study will inform the domestication, improvement, and conservation of *H. compressa* by selecting elite accessions.

## 1. Introduction

*Hyphaene compressa* (doum palm) H. Wendl. is a common palm in East Africa [1, 2]. It belongs to the Coryphoideae subfamily of the Arecaceae family [3]. The genus *Hyphaene* also known as the “doum palms” is predominant in Africa and has eight species, namely, *H. compressa, H. guineensis* Schumach. & Thonn*, H. coriacea* Gaertn*., H. macrosperma* H. Wendl., *H. reptans* Becc*., H. petersiana* Klotzsch ex Mart*., H. dichotoma* Furtado, and *H. thebaica* (L.) Mart. distributed in dry regions of Africa, Arabia, and India [2, 4]. In Africa, the genus *Hyphaene* has a wide range of uses that include but are not limited to the source of non-timber products for construction materials, food, medicine, and woven products as documented by several studies [1, 5, 6].

Despite the important economic role and contributions the genus makes to the palm family diversity in Africa, the genus is still poorly understood and evaluated [4, 7]. Of concern is the steady decline of doum palm populations in Africa due to destruction of their cradle habitat, drought, and overharvesting, thereby exacerbating pressure on the remaining African doum palm accessions which could inevitably lead to loss of their gene pool [4].

In Kenya, *H. compressa* plays a significant role in the livelihoods of people especially the pastoralist communities who rely on it for food, construction materials, medicine, and income through the sale of woven products [6]. The most important use of *H. compressa* in Kenya is food. There is increasing interest in doum palm domestication in some of the arid and semi-arid regions of Kenya. The drive for this is the decline in doum palm germplasm resources in these areas due to human interference and biotic stress [8]. *H. compressain situ* conservation is found in six protected areas and five *ex situ* conservation areas globally [9, 10]. In Africa, *in situ* conservation status of doum palms is limited and difficult to ascertain [11]. Moreover, the IUCN red list has categorized doum palm as a least concern species and is not yet in the category of near-threatened species. This could be the reason for the limited conservation efforts in the region. However, increased anthropogenic activities might lead to loss of doum palm biodiversity and ultimately more conservation efforts will be advocated for in the future. Therefore, conservation and diversity studies are needed to hasten the process of domestication and genetic improvement.

Diversity can be assessed using morphological variations which are informative enough for evaluation and description [12, 13]. Phenotypic characterization is the basic step for the classification, conservation, and utilization of genetic resources [14]. The present lack of knowledge on *H. compressa* limits access to its important traits and hence a hindrance to its improvement. Besides, it has no model descriptors which can aid in diversity studies. It, therefore, has no reference values at the International Plant Genetic Research Institute (IPGRI). It is important to determine the unique phenotypic descriptors for *H. compressa* which can be relied upon to distinguish members of this group [15]. A descriptor is a collection of standardized features used to provide information for describing and classifying a specific group of genetic resources [16]. According to IPGRI (https://www.bioversityinternational.org/e-library/publications/descriptors/), to facilitate international exchange and use of genetic resources uniformly, it is important to standardize these descriptors. Other palms like coconut, sago palm, peach palm, and date palm have descriptors that can be assessed at the IPGRI website. Morphological diversity study is the initial step for plant breeding. Therefore, to enhance doum palm, the diversity of its morphology is important. The objectives of this study were to determine the morphological variability of the vegetative and fruit traits of *H. compressa* and to identify the morphotypes of doum that are important for its improvement. It is assumed that the vegetative and fruit traits of doum palm are important in doum palm phenotypic diversity. It is also assumed that there are different morphotypes of doum palm.

## 2. Materials and Methods

### 2.1. Study Area

**Figure 1:**
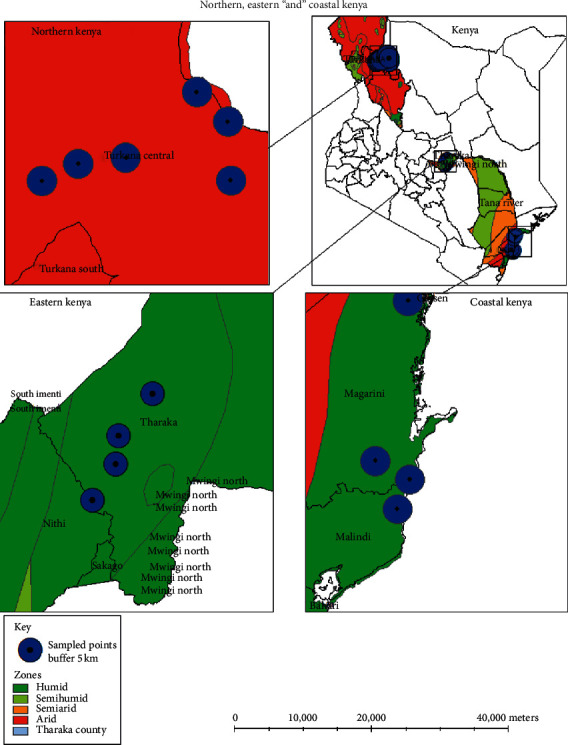
Sampling points in the different counties of Kenya from which *Hyphaene compressa* (doum palm) accessions were collected in 2018.

This study was done in three regions of Kenya: Northern (Turkana County), Eastern (Tharaka Nithi County), and Coastal (Tana River and Kwale County) as shown in Figure 1. These regions are characterized by high temperatures ranging from 20°C to 41°C and erratic rainfall of 280 mm to 2200 mm. The attributes of each of these study areas are summarized in Table 1.

### 2.2. Sampling

Sampling was done between January and July 2018 when doum palm trees were fruiting. Identification of doum palm was done with the aid of a taxonomist from the National Museums of Kenya. The selection criteria included the gender of the plant, the maturity of the tree, and the general good health of the palm and fruits. Only fruiting palms were selected for morphological diversity study. This is because distinguishing the nonflowering males from nonfruiting females is difficult in the wild populations. Moreover, doum palm has limited descriptors that can aid in diversity studies; therefore, fruit traits are important which are lacking in the male. Purposive sampling was used to select 30 female trees from each region. Doum palm trees sampled were separated from each other by at least 200 meters to reduce the probability of sampling close relatives [22]. From each sampled female tree, 10 fruits were randomly collected. The collected fruits were labelled, placed in bags, and transported to the laboratory for morphological assessment. The fruits collected from each tree were pooled and stored in one bag [22]. Some of the descriptors used for morphology were adapted from a descriptor list available for date palm [16]. All collected fruits were cleaned in running sterile water and left in the open to dry in the sun [22]. This was followed by the assessment of fruit morphological descriptors (Table 2). Fruit length and width were measured using vernier calipers [23]. The fruit weight was measured using an electronic weighing scale (Sartorius Entris 64-1S).

Some of the morphological parameters used in this study included those used by Rizk and El Sharabasy [16]. The morphology of the leaves and stem was assessed in the field during sampling. Leaf morphological characters were assessed as an average of five well-developed doum palm leaves [24]. Quantitative and qualitative vegetative traits were recorded (Table 2). Photographs of the plant leaves, stem, and fruits were taken to document their morphology and any differences were noted.

### 2.3. Data Analysis

The mean, range, and coefficient of variation were calculated for quantitative traits per sampled region. The frequencies for qualitative data were also recorded. The analysis of variance (ANOVA) was performed to determine the difference in the mean among categories and sites [25]. The Games–Howell Post Hoc Test was used to determine specifically which two treatments differed significantly from each other for the different phenotypic traits. Standardization of data was done because different scales of measurement were used for the different quantitative parameters assessed [14, 26]. Linear mixed-effects model using Ime4 package in *R* was used to assess the morphological diversity of doum palm according to the geographical regions of collection. The principal component analysis (PCA) using prcomp package in *R* was done to identify the most discriminating traits among the sampled sites. Discriminant analysis was done to estimate and describe each population using the MASS package in R. All the quantitative data were standardized prior to discriminant analysis. Clustering was done using Gower distance with the PAM (Partitioning around Medoids) algorithm using the daisy package in R. Both the numeric and categorical data were used for the cluster analysis. The silhouette coefficient was used to determine the number of clusters. All the statistical analyses were done in *R* version 4.0.2.

## 3. Results

### 3.1. Morphological Diversity of Fruit and Vegetative Traits

The frequencies of the quantitative traits are summarized in Table 3. There was high variability for doum palm height (cv = 38.3%). The fruit sizes ranged from 48.2 g to 148.8 g (cv = 21.5). There was low variability in the fruit length (cv = 11.8). There was variability in the fruit and vegetative quantitative traits of *H. compressa* per region (Table 4). All the seven quantitative traits were able to effectively discriminate doum palm phenotypically (*p* ≤ 0.001; Table 4). There was no significant difference in the quantitative traits of doum palm between Kwale and Turkana for leaf length, leaf breadth, fruit length, and fruit weight. Tharaka Nithi had the highest mean height (13.5 m) with the least being Kwale (5.65 m). The leaf breadth was significantly smaller (*p* ≤ 0.001) in Tana River (55.87 cm) than the other sampling sites. Tana River had the highest mean leaf length (120.2 cm) and fruit length (7.64 cm) with a *p* value of 0.000473 and <6.32*e* – 12, respectively.

There was a positive correlation between doum palm height and leaf length (*p* ≤ 0.001), leaf breadth (*p*=0.006), fruit breadth (*p*=0.029), fruit weight (*p* ≤ 0.001), and fruit length (*p* ≤ 0.001). There was a negative correlation between petiole length and all the quantitative fruit traits, fruit length (*p* ≤ 0.001), fruit breadth (*p*=0.004), and fruit weight (*p* ≤ 0.001) as shown in Table 5.

A linear mixed-effects model was fitted to predict *H*. *compressa* fruit weight with height, leaf length, leaf breadth, petiole length, fruit length, and fruit breadth. The model included the four sampling regions as random effects. The model's total explanatory power was substantial (conditional *R*^2^ = 0.80), and the part related to the fixed effects alone (marginal R2) was 0.66. The model's intercept was at -70.25. Within this model, the effect of fruit length on fruit weight was significant (beta = 19.01, std. beta = 0.68, *p* ≤ 0.001) and the effect of fruit breadth on fruit weight was significant (beta = 7.35, std. beta = 0.16, *p* < 0.05). The effects of height, leaf length, leaf breadth, and petiole length were not significant.

**Figure 2:**
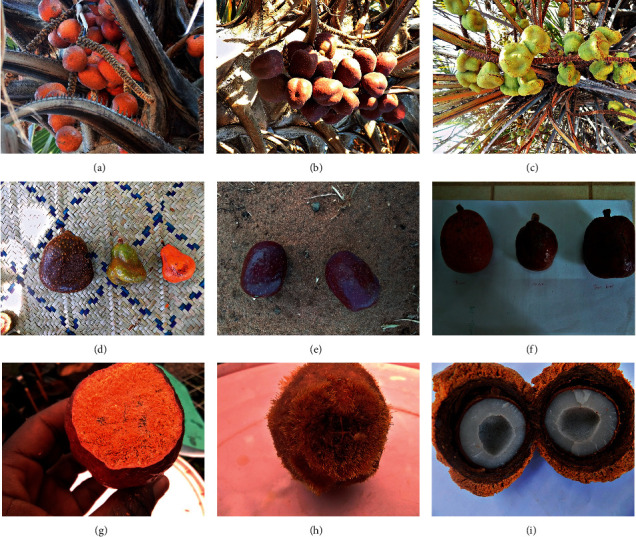
*H. compressa* fruit morphology in Kenya. (a) Mature doum palm fruits that are orange in colour in Tharaka. (b) Immature doum palm fruits maroon in colour in Turkana. (c) Immature doum palm fruits green in colour in Kwale. (d) Fruit morphology in Kwale: from left to right: round oblong shape (brown in colour), ovate shape (green), and obovate shape (orange). (e) Immature doum palm fruits maroon in colour. (f) From left to right: fruits from Tharaka Nithi, Turkana, and Tana River. (g) Orange mesocarp of the doum palm fruit. (h) Hairy mesocarp of doum palm fruit. (i) Cross section of the doum palm fruit.

Qualitative fruit and vegetative traits showed greater variability in trunk branching, mature fruit colour, trunk colour, leaf colour, and trunk diameter whereas little diversity was seen in terms of fruit shape, fruit apex shape, fruit base shape, mid-rib colour, unripe fruit colour, and petiole colour. All the doum palm fruits sampled had shiny skin which was fused with the flesh. The mesocarp was orange in colour and fibrous in texture with a characteristic strong aroma (Figures 2(a), 2(g), 2(h), and 2(i)). All the fruits sampled from Tharaka Nithi and Turkana were oblong shaped with truncate bases and apices (Table 6).

The fruits from Kwale showed the most diverse traits with differing shapes, bases, and apices (Figure 2(d)). The colour of unripe doum palm fruits was green in Kwale (Figure 2(c)), Tana River, Tharaka Nithi, and partly in Turkana. A total of 43.3% of the fruits sampled from Turkana were maroon when unripe (Figure 2(b), Table 6). The colour of mature doum palm fruits differed across the four sampling sites with the majority of the fruits being reddish-brown. All the fruits sampled from Tana River were reddish-brown when ripe while the fruits sampled from Tharaka Nithi were either brown (30%), orange-brown (63.3%), or orange (6.7%) as shown in Table 6.

**Figure 3:**
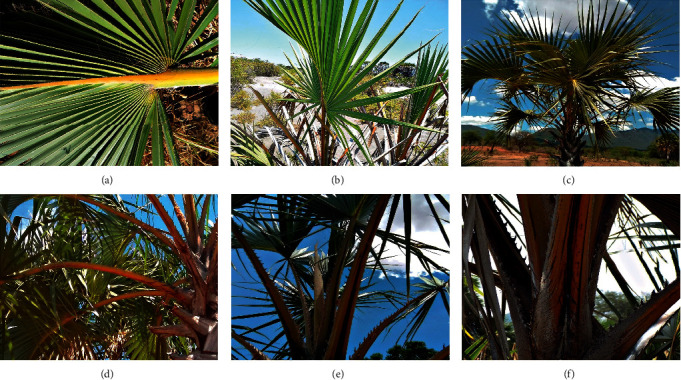
*H. compressa* leaf and petiole morphology. (a–c) Curved costa of *H. compressa*. (d) Long thin petioles observed in Kwale. (e) Petiole colour (yellow with black stripe) in Tana River. (f) Stout leaf base morphology was observed in all *H. compressa* trees.

**Figure 4:**
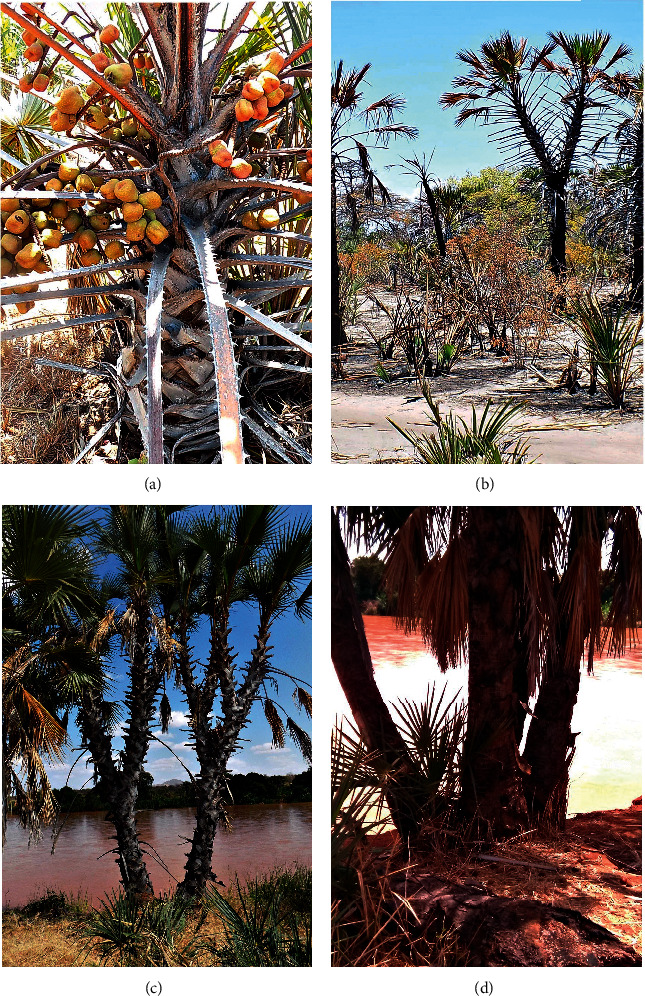
Branching morphology of *H. compressa*: (a) Single-trunk morphology showing low fruiting height in Kwale. (b) Two-trunk branching above the ground. (c) Two-trunk branching on the ground, middle, and top forming 8 crowns. (d) More than two-trunk branching at the ground level.

All leaf petioles were stouter at the base than at the top with varying petiole colours and curved costa (Table 6, Figure 3) The branching pattern observed in doum palm differed with some palms not branching at all. However, the majority of the palms had dichotomizing trunks. In Kwale, 46.7% of the sampled palms did not have any trunk branching. Two-trunk branching was common in all of the study sites with Tana River having the highest number of palm trees with two-trunk branching (80%) as shown in Table 6. On the other hand, Turkana and Tharaka had 10% and 33.3%, respectively, of the sampled doum palm trees with more than 2-trunk branching (Table 6). Trunk branching was either at the base (Figure 4(d)) or mid-section (Figures 4(b) and 4(c)).

### 3.2. Relationships between Discriminant Morphological Descriptors

The following discriminant models were derived:

(1) $$\begin{array}{c} \text{LD}1=0.55\text{ Ht}-0.0009\text{ LL}+0.47\text{ LB}-0.47\text{ PL}+1.55\text{ FL}-0.52\text{ FB}+1.19\text{ FWGT,}\\ \text{LD}2=0.04\text{ Ht}-0.12\text{ LL}-0.24\text{ LB}+0.20\text{ PL}+0.92\text{ FL}+1.29\text{ FB}-0.79\text{ FWGTg,}\\ \text{LD}3=0.90\text{ Ht}-0.57\text{ LL}+0.80\text{ LB}-0.02\text{ PL}-1.42\text{ FL}+0.66\text{ FB}+0.40\text{ FWGT,} \end{array}$$

where LD1, LD2, and LD3 are discriminant functions, Ht is the height, LL is the leaf length, LB is the leaf breadth, PL is the petiole length, FL is the fruit length, FB is the fruit breadth, and FWGT is the fruit weight.

**Figure 5:**
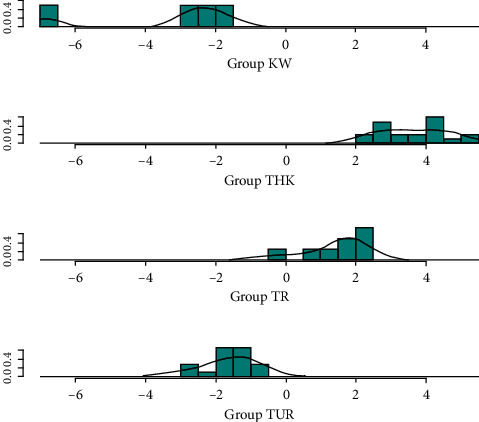
Separation between groups and overlapping areas that predict classes in doum palm using linear discriminant analysis. KW: Kwale. THK: Tharaka. TR: Tana River. TUR: Turkana.

LD1 explained 76.2% of the variation while LD2 and LD3 explained 15.03% and 8.8%, respectively. The second and third factors do not contribute much to discriminating between the groups. There were samples within Kwale that did not show any overlap with any of the groups from Tharaka, Turkana, and Tana River. There was an overlap of samples between Turkana and Kwale and between Tana River and Tharaka (Figure 5).

### 3.3. Principal Component Analysis

**Figure 6:**
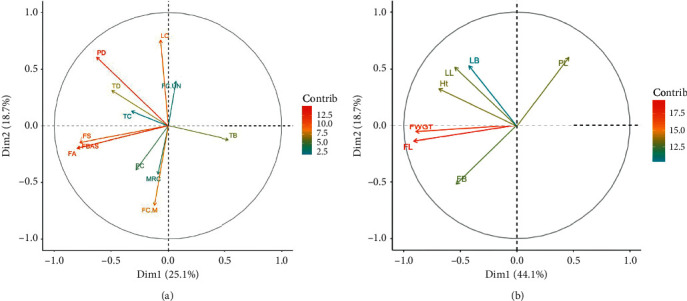
Variables' PCA plot of quantitative and qualitative traits of *H. compressa* in Kenya. (a). Variable PCA plot of qualitative traits. FS: fruit shape, FA: fruit apex, FBAS: fruit base, FC.UN: fruit colour-unripe, FC.M: fruit colour-mature, TC: trunk colour, TD: trunk diameter, TB: trunk branching, LC: leaf colour, MRC: mid-rib colour, PC: petiole colour, and PD: pinnae density. (b). Variables PCA of quantitative traits. H: height, LL: leaf length, LB: leaf breadth, PL: petiole length, FL: fruit length, FB: fruit breadth, and FWGT: fruit weight.

The first, second, and third components explained up to 59% of the variability in doum palm qualitative traits (Table 7). Component 1 explained 25% of the variability which was positively correlated with fruit shape, fruit apex, fruit base, trunk diameter, and pinnae density and negatively correlated with fruit colour when unripe and trunk branching (Figure 6(a)). The second component explained 18% of the variability which was positively correlated with leaf colour while the third component explained 15% of the variability correlated with trunk colour and trunk branching.

The first, second, and third components explained 75% of the variability in the quantitative traits (Table 8). The first component explained 44% of the variability which was correlated with fruit length, fruit breadth, fruit weight, height, and leaf length. The second component explained 19% of the variability related to leaf breadth and petiole length (Table 8).

Component 1 was negatively correlated with petiole length and positively with all the fruit traits, leaf length, width, and tree height; that is, the bigger the fruit, the shorter the petiole. Component 2, on the other hand, was negatively correlated with fruit characteristics and positively correlated with vegetative data (Figure 6(b)).

**Figure 7:**
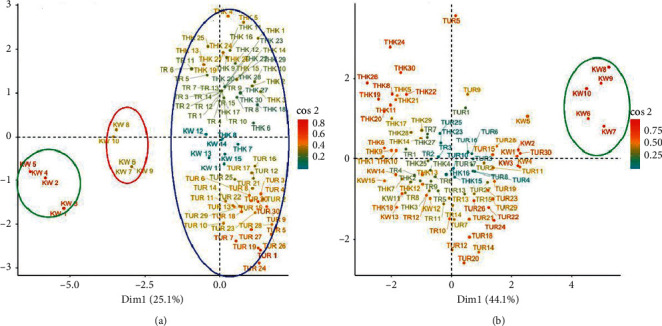
Individual accessions' PCA for qualitative and quantitative traits in *H. compressa* in Kenya. (a) Individuals' PCA using qualitative traits showing three clusters. (b) Individuals' PCA using quantitative traits showing two major clusters. KW: Kwale, THK: Tharaka Nithi, TR: Tana River, and TUR: Turkana.

**Figure 8:**
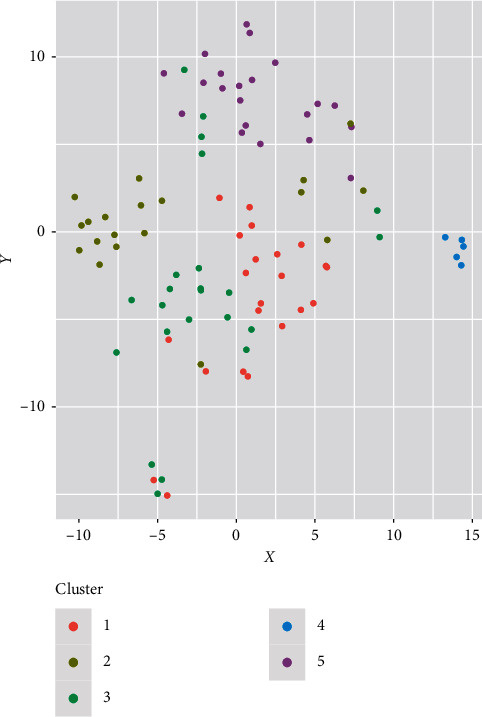
Cluster analysis in a lower-dimensional space of *H. compressa* (duom palm) accessions from Kenya based on quantitative and qualitative traits using Gower distance analysis. The morphotypes are colour coded.

Individual PCA based on qualitative and quantitative traits clustered the doum palm into three and two major clusters, respectively (Figures 7(a) and 7(b)). Five samples from Kwale clustered on their own using both qualitative and quantitative traits. The same samples also formed their own cluster after hierarchical clustering and are represented as morphotype 4 (Table 9, Figure 8).

### 3.4. Cluster Analysis

The hierarchical clustering of quantitative traits of the 90 doum palm samples clustered the samples into 5 morphotypes (Table 9, Figure 8). Morphotype 1 had 77.3% of doum palm from Turkana. All the doum palms belonging to morphotype 4 were from Kwale. A total of 90.5% of the palms belonging to morphotype 5 were from Tharaka. Morphotype 3 had representative palms from the four sampled regions of Kenya (Table 9). Some of the sampled palms from Kwale clustered with morphotypes 1, 3, and 5 indicating that these palms in Kwale are heterogeneous.

### 3.5. Identification of Elite Doum Palm

The minimum, maximum, and mean of the morphological traits are shown in Table 9. Morphotype 5 had the tallest trees (mean = 14) with the biggest fruits (mean = 129.4). Members of this cluster include palms from Tharaka (90.5%) and Kwale (9.5%). Tharaka samples that clustered together showed close homogeneity. Morphotypes 2 and 3 showed intermediate fruit sizes and traits. In addition, morphotype 2 had the longest leaves. Morphotype 4 had the shortest palms (mean = 3.96) with the smallest fruits (mean = 53.62) and the longest petioles (mean = 141.6). Morphotype 5 should be selected for improvement due to its fruit traits.

## 4. Discussion

High diversity was observed in quantitative traits within the individual doum palm trees sampled as well as among the different geographical sites sampled. There was also a high variability in the fruit qualitative traits with Kwale having the most diverse fruits. The mature fruits varied from reddish-brown, brown, to orange. Doum palm fruits are mostly green when unripe but later mature to orange, brown, red, or yellow [7]. However, other studies have reported that the colour of mature fruits tends to be orange-brown in colour [27]. In this study, the fruit weight varied from 48.2 g to 148.8 g. Another study on the update of the African palms noted that fruit size seemed to be greater in areas with no water stress [7]. The small-sized fruits in Turkana could possibly be explained by phenotypic plasticity due to resource limitation [28]. However, fruit sizes in Kwale varied from very large (morphotype 5) to very small (morphotype 4), which could be a result of belonging to different varieties. According to Stauffer et al. [2]. *Hyphaene compressa* fruits are extremely polymorphic with green immature fruits which turn to orange-brown at maturity. This indicates that the fruits in Kwale are heterogeneous. The analysis of variance of all the quantitative traits evaluated in this study was significant. This is similar to a study done to assess the phenotypic and molecular diversity in *H. thebaica,* where the authors reported significant differences in all the phenotypic traits evaluated. That study further indicated that phenotypic and molecular analyses were complementary to each other in evaluating *H. thebaica* even though they gave different relationships among the samples tested [26].

The PCA clustering of the samples using both quantitative and qualitative traits indicates that two major clusters are formed with a subset of samples from Kwale clearly forming their own cluster from the rest of the accessions. These five accessions seem to be distantly related to the others. These samples also seem not to show any overlap with any samples from the other regions based on linear discriminant analysis. However, this cannot be used to delineate this group since advanced markers would be required to genotype it [13]. Most palm species have cylindrical, elongated, and unbranched stems. *H. compressa,* on the other hand, has dichotomizing trunks which is a unique feature for *Hyphaene* where the basal stem is overbuilt to handle the later dichotomous branching [29]. However, 46.7% of the accessions from Kwale did not have dichotomizing trunks. This suggests variability at the Kenyan Coast especially in Kwale compared to the rest of the regions.

The representation of the different samples in different clusters indicated that doum palm is genetically diverse. Therefore, the different morphotypes identified in this study might not be directly influenced by their environment. This is supported by Gower cluster analysis and projection of the samples on PCA which indicated a high level of heterogeneity. Cluster analysis in this study revealed phenotypic diversity and heterogeneity within samples from the same region. For instance, accessions from Kwale were clustered in morphotypes 1, 3, and 5. Kwale also had some accessions forming a lone cluster, the morphotype 4. They were, therefore, the most diverse with some accessions having very tall trunks with very large fruits while others were very short with small fruits. This heterogeneity was also observed among accessions from Tharaka and Turkana with fruits from each region clustering into three different morphotypes. This heterogeneity within samples from the same region has been previously reported [13].

Identification of the different morphotypes in existence will help farmers and stakeholders to identify specific accessions for their own use, improvement, and conservation. There are no known improvement strategies for doum palm. Farmers in Tharaka prefer a specific doum palm for weaving because the leaves are longer and wider than the other trees. Such information can help breeders select traits for improvement and mass production. The present study noted that the longest and widest leaves used for weaving were found in Tharaka (90.5%) and Kwale (morphotype 5). The fact that Turkana which is the most arid region of all the sampled areas had accessions that form long and wide leaves just like in Tharaka, which receives a slightly higher amount of rainfall than Turkana, suggests that the difference in leaf lengths and breadths might not be influenced by the environment in *H. compressa.* It is indeed in these regions (Tharaka and Turkana) where massive weaving is done using doum palm leaves. If the morphotypes are superior for a specific trait that is desired by farmers, then it is only prudent that they are selected for improvement/breeding. In this study, morphotype 5 had the biggest fruits and can be selected for improvement.

*H. compressa* has costapalmate, fan-shaped leaves with entire margins, curved costa, and curved thorns on the leaf stalk [30]. The petiole length seemed to be an important trait in discriminating this palm. Petiole length was significantly longer in morphotype 4. There was a negative correlation between the petiole length and fruit traits. That is, the bigger the fruits, the shorter the petiole and vice versa. Petioles are important resources for the local communities especially the nomadic-pastoralists of Kenya who use them for furniture and construction of houses [6]. Significantly, longer petioles on shorter trees are important for construction. Local communities would then prefer this accession for this purpose. The present study also reports that morphotype 4, in spite of having longer petioles, is a short accession. This advantage will benefit the users by making the petioles easily accessible compared to taller palms. Morphological diversity is of benefit to preliminary doum palm genetic resource evaluation. However, the superior traits identified cannot adequately resolve the differences in diversity and should be confirmed if indeed they are genetically determined. The diversity of *H*. *compressa* could be further investigated by genome-wide associations or other next-generation approaches. The main limitation of this study is the exclusion of male doum palms. Additionally, few descriptors were used in the present study as doum palm has no known standard descriptors. Future studies should evaluate additional descriptors so that the male doum palm diversity can also be determined.

## 5. Conclusion

This study assessed the variability in morphological traits of *H. compressa* and identified its morphotypes. The results show that there was variability in the fruit and vegetative traits of *H. compressa* per region. This study identified five morphotypes of *H. compressa* from the Northern, Coastal, and Eastern regions of Kenya. Different morphotypes showed superior traits for fruits, leaves, and petioles making it possible to select superior accessions for domestication and genetic improvement. Morphotype 5 should be considered for the improvement of leaf and fruit traits.

## Figures and Tables

**Table 1:** Attributes of the study sites from which *Hyphaene compressa* (doum palm) samples were collected in Kenya in 2018.

Region	Coastal		Eastern	Northern
	Kwale	Tana River	Tharaka Nithi	Turkana
Coordinates	38° 31 and 39° 31׳ east	38° 25׳ and 40° 15׳ east	37° 19׳ and 37° 46׳ east	34° 30׳ and 36° 40׳ east
	30°3׳ and 40° 45׳ south	0° 0׳ and 2° 0׳ south	00° 07׳ and 00° 26׳ south	1° 30׳ and 5° 30׳ north
Total area (km^2^)	8270	38682	2662	77,000
Rainfall (mm)	400–1680	280–900	500–2200	300–450
Temperature (°C)	24–36	23–38	14–36	20–41
Major economic activities	Tourism, fishing agriculture mining (titanium)	Agri-pastoralists fishing	Agri-pastoralism fishing	Pastoralism, fishing

Sources [17–21].

**Table 2:** Quantitative and qualitative descriptors used for the morphological characterization of doum palm.

Plant part	Descriptor	
	Quantitative traits	Qualitative traits
Whole plant	Height (in m)	

Trunk		Colour (dark brown, pale, ashy) Diameter (thick, medium, thin), Branching pattern (None, 2 trunk branching, more than 2)

Leaves	Length (cm), Breadth (cm), petiole length (cm),	Colour (dark green, green, light green) Mid-rib colour (green, yellow green) Pinnae density (very dense, dense, lax) Petiole colour (green with black stripes, green, light green, yellow with black stripes, brown with black stripes)

Fruit	Length (cm), width (cm), weight (gm)	Shape (round oblong, obviate, ovate), Fruit apex (truncate, depressed), Fruit base (truncate, acute), Unripe fruit colour (green, maroon), Mature fruit colour (reddish-brown, brown, orange-brown, orange)

Reference [16].

**Table 3:** Frequency distribution table of the quantitative morphological traits used for the morphological characterization of doum palm.

Descriptor (Unit of measurement)	Range	Mean	CV (%)
Height (m)	1.8–20	10.15	38.3
Leaf length (cm)	61–161	106.6	22.1
Leaf breadth (cm)	30–124	74.57	25.7
Petiole length (cm)	52–153.1	97.64	20.1
Fruit length (cm)	4.7–8.4	7.05	11.8
Fruit breadth (cm)	4.4–7.1	6.1	8.1
Fruit weight (g)	48.3–148.8	107.21	21.5

**Table 4:** Mean for quantitative traits of doum palm in sampled regions of Kenya.

Trait	Tharaka	Tana river	Kwale	Turkana	*p* value
	Mean ± se	Mean ± se	Mean ± se	Mean ± se	
Height (H)	13.5 ± 3.73a	9.93 ± 2.58b	5.65 ± 2.28c	9.16 ± 1.94b	8.24*e* − 13^*∗∗∗*^
Leaf length (LL)	114.73 ± 26.25a	120.2 ± 22.78a	96.2 ± 11.72b	96.93 ± b	0.000473^*∗∗∗*^
Leaf breadth (LB)	89.47 ± 13.12a	55.87 ± 7.73c	73.33 ± 12.53b	69.63 ± 20.3b	3.24*e* − 09^*∗∗∗*^
Petiole length (PL)	92.47 ± 12.06b	93.27 ± 13.7ab	109 ± 24.94a	99.33 ± 23.38ab	0.0426^*∗*^
Fruit length (FL)	7.59 ± 0.34a	7.64 ± 0.3a	6.33 ± 1.36b	6.56 ± 0.25b	6.32*e* − 12^*∗∗∗*^
Fruit breadth (FB)	6.031 ± 0.227a	6.27 ± 0.228a	5.6 ± 0.798b	6.32 ± 0.40a	6.36*e* − 06^*∗∗∗*^
Fruit weight (FWGT)	127.6 ± 11.24a	111.53 ± 9.3b	91.73 ± 34.94c	92.37 ± 9.11c	2.09*e* − 12^*∗∗∗*^

Same letters within the row indicate no significant difference between the means while different letters indicate a significant difference between the means at *α* = 5% significance codes ^*∗*^ = 0.01 and ^*∗∗∗*^ = 0.000. The Games–Howell post hoc test was used for multiple comparison.

**Table 5:** Correlation between quantitative traits of doum palm from Kenya.

	Ht	LL	LB	PL	FL	FB
LL	0.425^*∗∗*^					
LB	0.288^*∗∗*^	0.270^*∗∗*^				
PL	−0.082	0.031	−0.073			
FL	0.501^*∗∗*^	0.415^*∗∗*^	0.247^*∗*^	−0.425^*∗∗*^		
FB	0.231^*∗*^	0.121	−0.056	−0.300^*∗∗*^	0.476	
FWGT	0.521^*∗∗*^	0.345^*∗∗*^	0.346^*∗∗*^	−0.378^*∗∗*^	0.861^*∗∗*^	0.386^*∗∗*^

^*∗∗*^Correlation is significant at the 0.01 level. ^*∗*^Correlation is significant at the 0.05 level. H: height, LL: leaf length, LB: leaf breadth, PL: petiole length, FL: fruit length, FB: fruit breadth, and FWGT: fruit weight. Correlation analysis using Pearson's correlation.

**Table 6:** Fruit and vegetative qualitative traits of Kenyan doum palm accessions.

Trait	Category	Tharaka %	Tana River %	Kwale %	Turkana %
Trunk colour	Dark brown colour	16.7	—	—	10
	Pale colour	26.7	100	—	6.7
	Ashy colour	56.7	—	100	88.3

Trunk diameter	Thick	53.3	—	26.7	16.7
	Medium	46.7	80	40	88.3
	Thin	—	20	33.3	—

Trunk branching	No branching	—	13.3	46.7	6.7
	2-trunk branching	66.7	80	40	60
	More than 2 branching	33.3	6.7	13.3	10

Leaf colour	Dark green	36.7	—	—	6.7
	Green	63.3	—	100	46.7
	Light green	—	100	—	46.7

Mid-rib colour	Green	—	—	—	23.3
	Yellow green	100	100	100	76.7

Petiole colour	Green with black stripes	33.3	—	6.7	80
	Green	16.7	—	26.7	16.7
	Yellow with black stripes	20	100	—	—
	Brown with black stripes	30	—	66.7	3.3

Pinnae density	Very dense	100	—	—	3.3
	Dense	—	100	53.3	96.7
	Lax		—	46.7	—

Fruit shape	Round oblong	100	93.3	33.3	100
	Obviate	—	6.7	33.3	—
	Ovate	—	—	33.3	—

Fruit apex	Truncate	100	100	66.7	100
	Depressed	—	—	33.3	—

Fruit base	Truncate	100	100	33.3	100
	Acute	—	—	66.7	—

Fruit colour-unripe	Green	100	100	100	56.7
	Maroon	—	—	—	43.3

Fruit colour when mature	Reddish-brown	—	100	6.7	56.7
	Brown	30	—	60	43.3
	Orange-brown	63.3	—	—	—
	Orange	6.7	—	33.3	—

**Table 7:** Principal component analysis of qualitative traits of doum palm in arid and semi-arid lands of Kenya.

	Principal components											
	1	2	3	4	5	6	7	8	9	10	11	12
FS	**0.77**	−0.15	0.40	−0.16	−0.18	0.11	0.04	0.32	−0.01	0.09	−0.19	0
FA	**0.80**	−0.20	−0.40	0.33	0.10	0.11	−0.14	−0.04	0.09	−0.03	0.02	0
FBAS	**0.80**	−0.20	−0.40	0.33	0.10	0.11	−0.14	−0.04	0.09	−0.03	0.02	0
FC.UN	−**0.07**	0.39	0.42	0.36	0.63	0.11	−0.06	−0.23	−0.04	0.23	−0.08	0
FC.M	0.12	−0.70	0.61	0.01	0.00	0.02	0.00	0.11	0.10	0.26	0.17	0
TC	0.32	0.13	**0.35**	0.56	−0.16	−0.55	0.30	−0.06	0.10	−0.09	−0.01	0
TD	**0.49**	0.31	0.40	−0.38	−0.03	0.37	0.25	−0.29	0.20	−0.15	0.04	0
TB	−**0.53**	−0.12	−**0.35**	0.41	0.10	0.38	0.43	0.23	0.12	0.03	−0.01	0
LC	0.07	**0.76**	−0.25	−0.27	0.15	−0.21	−0.07	0.25	0.35	0.15	0.03	0
MRC	0.09	−0.43	0.09	−0.29	0.76	−0.18	0.09	0.17	−0.01	−0.25	0.00	0
PC	0.28	−0.39	−0.58	−0.39	0.06	−0.23	0.29	−0.26	−0.04	0.26	−0.04	0
PD	**0.63**	0.61	−0.07	0.00	0.07	0.05	0.19	0.18	−0.37	0.05	0.12	0

Proportion of variance												
	0.25	0.18	0.15	0.11	0.09	0.06	0.04	0.04	0.03	0.03	0.01	0

Cumulative percentage												
	0.25	0.43	0.59	0.69	0.79	0.85	0.89	0.94	0.94	0.97	0.99	1

FS: fruit shape, FA: fruit apex, FBAS: fruit base, FC.UN: fruit colour-unripe, FC.M: fruit colour-mature, TC: trunk colour, TD: trunk diameter, TB: trunk branching, LC: leaf colour, MRC: mid-rib colour, PC: petiole colour, and PD: pinnae density.

**Table 8:** Variability of the principal component analysis of quantitative traits of doum palm in Kenya.

Trait	PC 1	PC 2	PC 3	PC 4	PC 5	PC 6	PC 7
H	**0.69**	0.33	0.21	0.23	−0.43	−0.36	0.01
LL	**0.55**	0.52	0.33	−0.49	0.26	−0.10	−0.04
LB	0.42	**0.53**	−0.60	0.26	0.32	−0.08	0.03
PL	−0.46	**0.60**	0.46	0.33	0.06	0.32	0.02
FL	**0.91**	−0.14	0.02	−0.07	−0.06	0.28	0.25
FB	**0.54**	−0.52	0.39	0.32	0.41	−0.15	−0.02
FWGT	**0.89**	−0.06	−0.10	0.05	−0.13	0.34	−0.23

Proportion of variance							
	0.44	0.19	0.13	0.08	0.08	0.07	0.02

Cumulative variance							
	0.44	0.63	0.75	0.84	0.92	0.98	1.00

PC: principal components, H: height, LL: leaf length, LB: leaf breadth, PL: petiole length, FL: fruit length, FB: fruit breadth, and FWGT: fruit weight.

**Table 9:** Quantitative traits of *H. compressa* morphotypes from arid and semi-arid lands of Kenya.

Number trait	Morphotype 1		Morphotype 2		Morphotype 3		Morphotype 4		Morphotype 5	
	22 (24.4%)		19 (21.1%)		23 (25.6%)		5 (5.6%)		21 (23.3%)	
	Mean	Range	Mean	Range	Mean	Range	Mean	Range	Mean	Range
Height	8.7	3–12	10	7–18	9.5	5–15	3.96	1.8–6	14	7–20
Leaf breadth	69	30–124	61.7	40–93	77.7	47–108	61.2	55–67	92.62	73–112
Leaf length	98.6	61–152	116.7	79–153	100.6	74–132	87.6	80–95	117	85–161
Petiole length	93.9	52–153	91.8	76–132	98.9	75–124	141.6	127–152	94.95	76–125
Fruit length	6.7	6–7.8	7.4	6.6–8.1	6.98	5.7–8.4	4.97	4.7–5.3	7.6	6.7–8.2
Fruit breadth	6.2	5.3–6.9	6.3	5.7–7.1	6.1	4.7–8.9	4	4.4–5.2	6.1	5.7–7.1
Fruit weight	95.7	80.8–125.6	112.9	97.1–136	104.95	75.4–148.6	53.62	48–63	129.4	109–149

Number (%) per sampling points										
Tharaka	4.5		21.1		26.1		0		90.5	
Tana River	4.5		57.9		13.0		0		0	
Kwale	13.6		0		21.7		100		9.5	
Turkana	77.3		21.1		39.1		0		0	

## Data Availability

All data generated or analysed during this study are included in this published article.
